# Short-term effects of transcranial direct current stimulation on motor speech in Parkinson’s disease: a pilot study

**DOI:** 10.1007/s00702-024-02771-5

**Published:** 2024-04-09

**Authors:** Lubos Brabenec, Daniel Kovac, Jiri Mekyska, Lenka Rehulkova, Veronika Kabrtova, Irena Rektorova

**Affiliations:** 1 Applied Neuroscience Research Group, Central European Institute of Technology – CEITEC, Masaryk University, Brno, Czech Republic; 2 Department of Telecommunications, Brno University of Technology, Brno, Czech Republic; 3 Faculty of Medicine and St. Anne’s University Hospital, First Department of Neurology, Brno, Czech Republic

**Keywords:** Parkinson’s disease, Hypokinetic dysarthria, Transcranial electric stimulation, Acoustic analysis, Superior temporal gyrus

## Abstract

Introduction: Hypokinetic dysarthria (HD) is a common motor speech symptom of Parkinson’s disease (PD) which does not respond well to PD treatments. We investigated short-term effects of transcranial direct current stimulation (tDCS) on HD in PD using acoustic analysis of speech. Based on our previous studies we focused on stimulation of the right superior temporal gyrus (STG) - an auditory feedback area. Methods: In 14 PD patients with HD, we applied anodal, cathodal and sham tDCS to the right STG using a cross-over design. A protocol consisting of speech tasks was performed prior to and immediately after each stimulation session. Linear mixed models were used for the evaluation of the effects of each stimulation condition on the relative change of acoustic parameters. We also performed a simulation of the mean electric field induced by tDCS. Results: Linear mixed model showed a statistically significant effect of the stimulation condition on the relative change of median duration of silences longer than 50 ms (*p* = 0.015). The relative change after the anodal stimulation (mean = -5.9) was significantly lower as compared to the relative change after the sham stimulation (mean = 12.8), *p* = 0.014. We also found a correlation between the mean electric field magnitude in the right STG and improvement of articulation precision after anodal tDCS (*R* = 0.637; *p* = 0.019). Conclusions: The exploratory study showed that anodal tDCS applied over the auditory feedback area may lead to shorter pauses in a speech of PD patients.

## Introduction

Approximately 90% of individuals with PD experience hypokinetic dysarthria (HD) as the disease progresses (Ramig et al. 2008). HD is defined by decreased variability in pitch and volume, imprecise articulation, impaired speech prosody, and inappropriate silences (Brabenec et al. 2017).

Studies examining the effect of dopaminergic medication on HD report mixed results (Brabenec et al. 2017). Most of the evidence suggests that levodopa has no significant short-term effects on speech (Skodda et al. 2010; Cavallieri et al. 2021). Longitudinal studies (Rusz et al. 2016; Tykalova et al. 2015) revealed that levodopa administration in the early stages of PD may improve consonant articulation but also lead to more dysfluent speech. A recent study (Rusz et al. 2021) found variable responses to long-term levodopa administration among early PD patients depending on specific HD phenotypes.

Speech findings after the deep brain stimulation (DBS) also vary (Skodda et al. 2014; Baudouin et al. 2023). Previous research showed that DBS may improve voice tremor or voice intensity (Tripoliti et al. 2011; Tsuboi et al. 2014), but at the same time, it may worsen speech intelligibility (Tripoliti et al. 2011, 2014; Pinto et al. 2014; Tsuboi et al. 2014). Predictive factors of this deterioration include longer disease duration and lower speech intelligibility before surgery (Tripoliti et al. 2014; Pinto et al. 2023). Taken together, the effects of pharmacological and surgical interventions on HD are limited and varied. Therefore, there is a need to explore other methods that target different aspects of speech and could potentially be used also in later stages of PD.

We focused our research on non-invasive brain stimulation methods (NIBS). In PD patients with HD, repetitive transcranial magnetic stimulation (rTMS) has been mostly applied over a primary orofacial area (OFSM1) with inconsistent results (Brabenec et al. 2017). However, previous research showed that particularly the right posterior superior temporal gyrus (STG), a cortical region involved in auditory speech feedback (Liu et al. 2023), plays an important role in modulation of motor aspects of speech production in PD patients (New et al. 2015; Klobusiakova et al. 2021). In our previous study (Brabenec et al. 2019), we demonstrated that a single session of 1 HZ rTMS over the right STG may lead to significant improvement of articulation in PD. The improvements were significantly higher than improvements after 10 Hz stimulation over the left OFSM1, and more pronounced than the stimulation of a control stimulation site (Brabenec et al. 2019). A subsequent study also showed that multiple sessions of active rTMS over the right STG, as compared to sham stimulation, had long-lasting positive effects on HD, particularly on perceptual measures of articulation, prosody, and speech intelligibility. These effects were supported by remote stimulation-induced brain plasticity changes within the articulatory networks (Brabenec et al. 2021).

In our current project, we aim to develop a program for remote treatment of HD. To achieve our goal, we chose transcranial direct current stimulation (tDCS) that can be used remotely by patients at home together with a concurrent Lee Silverman Voice Treatment (LSVT), i.e., the best documented therapy for symptoms of HD in PD (Yuan et al. 2020). In PD patients, tDCS and other modes of temporal electrical stimulation (tES) have mostly been used to modulate gait speed, freezing of gait, limb bradykinesia, attention and executive functions, and brain excitability (Ni et al. 2022). Studies also provided evidence that home-based tDCS treatment is feasible and safe for PD patients (Dobbs et al. 2018). To our knowledge, research on the effects of tDCS/ tES on HD symptoms in PD has not yet been conducted.

In this pilot study, we particularly concentrated on identifying a suitable electrode montage and examined immediate aftereffects of a single session of tDCS on HD symptoms. These acute effects could last up to an hour (Nitsche et al. 2008; Brunoni et al. 2012). Studies have also shown that different current flow directions may result in different aftereffects, and the direction of the excitability shift might be divergent, dependent not only on stimulation polarity, but also on the specific electrode montage (Nitsche et al. 2008). Therefore, we applied both anodal and cathodal tDCS to the right STG. We also used SimNIBS (Thielscher et al. 2015) software for simulation of the mean electric field induced by tDCS. Regarding behavioral outcomes, we focused on the acoustic parameters that were responsive to non-invasive brain stimulation methods in our previous studies (Brabenec et al. 2017, 2019).

## Methods

### Participants

The inclusion criteria for enrolment into the study were as follows: (1) clinically established PD (criteria by Postuma et al. 2016), (2) right-handedness, (3) presence of HD symptoms based on the assessment of a speech therapist and the results of a 3F Test Dysarthric profile total score (Kostalova 2013), (4) Czech as their first language. Exclusion criteria were (1) alcohol or drug abuse, (2) hallucinations, (3) any diagnosed psychiatric disorder (4) dementia, based on the Montreal Cognitive Assessment (MoCA) test for dementia, MoCA > 20 (Biundo et al. 2014), and on a clinician’s interview with a caregiver (5) cardio pacemaker or any MRI-incompatible metal in the body, (6) epilepsy.

The disease severity was assessed using the Unified Parkinson’s Disease Rating Scale (UPDRS), part III (Motor Examination) scale. All participants were on a stable dopaminergic medication at least 4 weeks prior to baseline assessment and during the whole study. The patients were tested in the ON medication state without dyskinesias since we wanted to modulate HD symptoms in a real-life scenario in patients on dopaminergic medication. All patients signed an informed consent form that was approved by the local ethics committee.

### Study design

Participants underwent tDCS over the right posterior STG. At the baseline visit, each participant underwent a speech assessment using the 3F Test Dysarthric profile (Kostalova 2013) (for details see Table S1 in Supplementary materials). Structural MRI scans (T1 MPRAGE) were performed for frameless stereotactic navigation of the electrode placement. After the baseline visit, each participant underwent three stimulation sessions (anodal, cathodal, and sham stimulation), separated by one day without stimulation. A crossover double-blind design was used, and stimulation protocols were randomized across subjects and sessions.

A protocol consisting of speech tasks lasted up to 10 min and was performed prior to and immediately after each stimulation session.

### Acoustic analysis of speech

The speech protocol contained a special reading task (reading a phonetically balanced paragraph containing 150 words; patients were allowed read the text in advance). HD symptoms were assessed using speech parameters of interest based on our previous research.(Brabenec et al. 2019) More specifically, we quantified tongue and jaw rigidity (relF1SD and relF2SD), monopitch (relF0SD), duration of silences (DurMED), and irregular speech rhythms (SPIR) (see Table 1 for detailed description of the parameters).

**Table 1:** Analyzed acoustic features in reading task

HD dimension and specific disorder	Acoustic feature	Feature definition	Feature interpretation
Rigidity of tongue and jaw	relF1SD, relF2SD	Standard deviation of first (F1) and second (F2) formant relative to its mean.	Higher value means better performance
Monopitch	relF0SD	Pitch variation, defined as a standard deviation of F0 contour relative to its mean.	Higher value means better performance
Irregular rhythm of speech	SPIR	Number of pauses relative to total speech time after removing periods of silence lasting less than 50 ms.	Higher value means better performance
Longer duration of silences	DurMED	Median duration of silences longer than 50 ms.	Lower value means better performance

### TDCS protocol

Stimulation was applied through a battery-driven device (DC-Stimulator Plus, neuroConn GmbH, Germany). Both electrodes were positioned over the right and left posterior superior temporal gyrus (STG) (MNI coordinates: X = 40, Y = − 38, Z = 14; X = − 40, Y = − 38, Z = 14; based on our previous research) (Brabenec et al. 2021). We used the T1 MRI scan-based frameless stereotactic neuronavigation to specify the exact location of the electrode center in each individual. For anodal stimulation, the anode was placed over the right STG and the cathode over the left STG. For cathodal stimulation, the cathode was placed over the right STG and the anode over the left STG.

A current of 2 mA was delivered using two rubber electrodes (5 × 5 cm) for 20 min. The electrode was held in place by a conductive gel. The sham stimulation was applied with the same settings, but the stimulator was turned off after 30 s.

### Statistical analysis

We used linear mixed models (LMM) to evaluate the effects of each stimulation condition on the relative changes in acoustic parameters. The stimulation condition was a fixed factor in LMM. Post-hoc pairwise comparisons of estimated marginal means were made with the Bonferroni correction. Age, gender, and levodopa equivalent dose (LED) were used as covariates in all LMMs. Wilcoxon signed-rank tests were used to compare the values of these parameters prior to and after each stimulation condition. A Spearman correlation analysis was used to assess associations between the tDCS-induced changes and the simulation of the electric field in right STG. These statistical procedures were performed with IBM SPSS Version 25.0 (IBM Corp., Armonk, NY, USA).

### Electric field simulation in SimNIBS

SimNIBS software (version 4.0.1) was used to calculate the electric field induced by tDCS, based on the finite element method and individualized tetrahedral head meshes generated from the structural T1 images of the participant. Electric field simulations were computed for both cathodal and anodal montages. Both electrodes were positioned based on the MNI coordinates mentioned previously. The mean electric field in the right STG (sphere radius = 10 mm) was calculated using a MATLAB script.

## Results

We enrolled 14 right-handed patients with clinically established PD. All had mild to moderate HD based on the assessment of a speech therapist and the results of a 3F Test Dysarthric total score (Kostalova 2013). The maximum total score is 90 (normal speech), and the minimum score is 0. See Table 2 for demographic and clinical data.

**Table 2:** Demographic and clinical variables

Gender Female/Male	7/7
**Age (years)**	Mean 70.78 (SD 7.84)
**Duration of PD (years)**	Mean 5.03 (SD 4.18)
**LED (mg/day)**	Mean 1014.04 (SD 343.16)
**UPDRS III**	Mean 11.21 (SD 4.24)
**3F Test Total score**	Mean 74.42 (SD 7.41)
**MOCA**	Mean 25.43 (SD 2.14)

M - Mean; SD - Standard deviation; PD - Parkinson’s disease; LED - Levodopa equivalent dose; UPDRS III - Unified Parkinson’s disease rating scale; MOCA - Montreal Cognitive Assessment

**Fig. 1:**
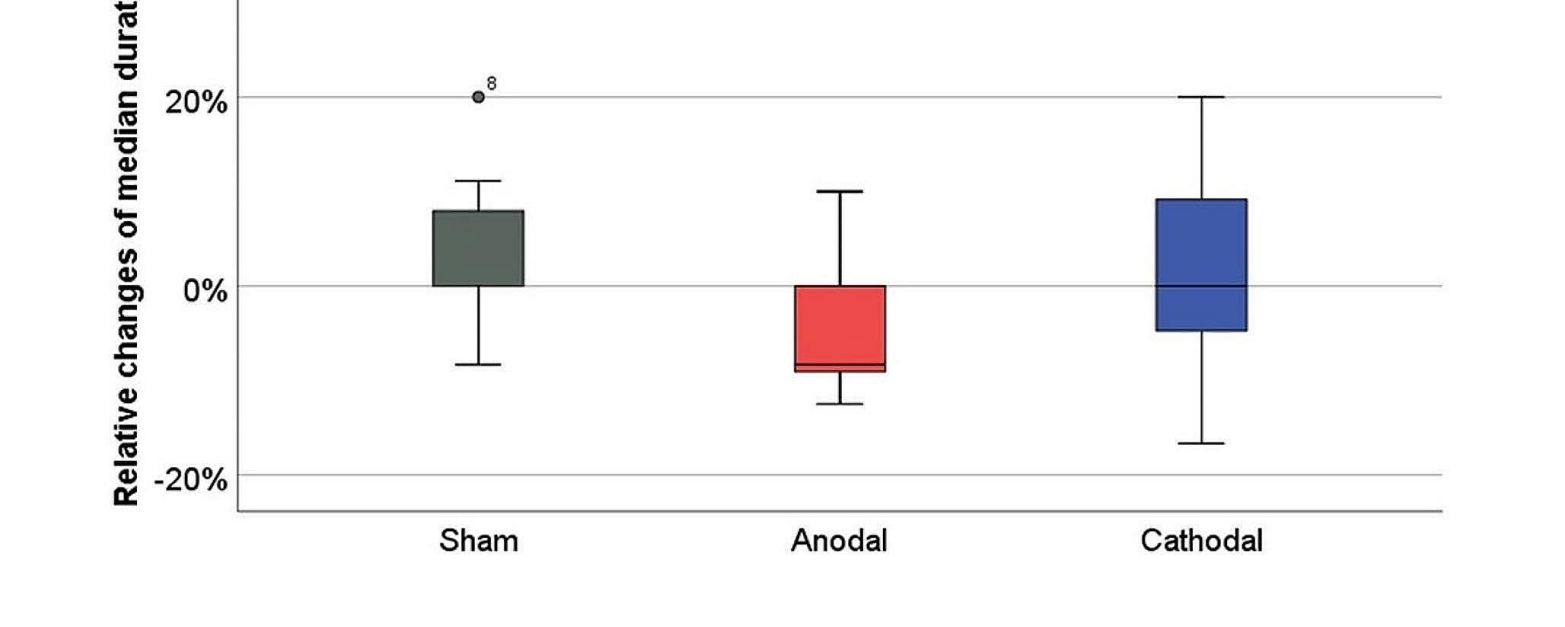
Relative changes in the median duration of silences longer than 50 ms after distinct active and sham tDCS

Using linear mixed model analysis (LMM) (see Table 3), we observed a significant effect of the stimulation condition on changes in the median duration of silences longer than 50 ms (F(2,21.8) = 5.1, *p* = 0.015), i.e. inappropriate silences that negatively impact speech rhythm and speech fluency (Brabenec et al. 2017). The relative decreases in long pauses after anodal stimulation (mean = -5.9) were significantly higher than the changes after the sham stimulation (mean = 12.8), *p* = 0.014, and non-significantly higher than the changes induced by the cathodal stimulation (mean = -0.5), *p* = 0.111 (see Fig. 1). Results of Post-hoc Wilcoxon test showed that anodal stimulation induced a significant decrease of this parameter (*p* = 0.047) (see Table 4).

**Table 3:** Acoustic analysis - Results of linear mixed models

Acoustic parameter	F	*p* value
relF1SD	5.136	0.211
relF2SD	2.528	0.104
relF0SD	1.657	0.214
SPIR	0.759	0.481
DurMED	5.135	**0.015**

**Table 4:** Results of Wilcoxon test; *p* values

	Acoustic parameter	Median before stimulation	Median after stimulation	*p* value
Anodal tDCS	relF1SD	0.560	0.586	0.463
	relF2SD	0.249	0.249	0.650
	relF0SD	0.162	0.160	0.087
	SPIR	2.011	1.913	0.861
	DurMED	0.110	0.110	**0.047**
Cathodal tDCS	relF1SD	0.589	0.559	0.064
	relF2SD	0.249	0.237	**0.039**
	relF0SD	0.156	0.158	0.311
	SPIR	2.031	1.834	0.087
	DurMED	0.105	0.100	0.670
Sham tDCS	relF1SD	0.579	0.557	0.136
	relF2SD	0.249	0.256	0.695
	relF0SD	0.170	0.166	0.272
	SPIR	1.991	1.971	0.638
	DurMED	0.110	0.110	0.127

**Fig. 2:**
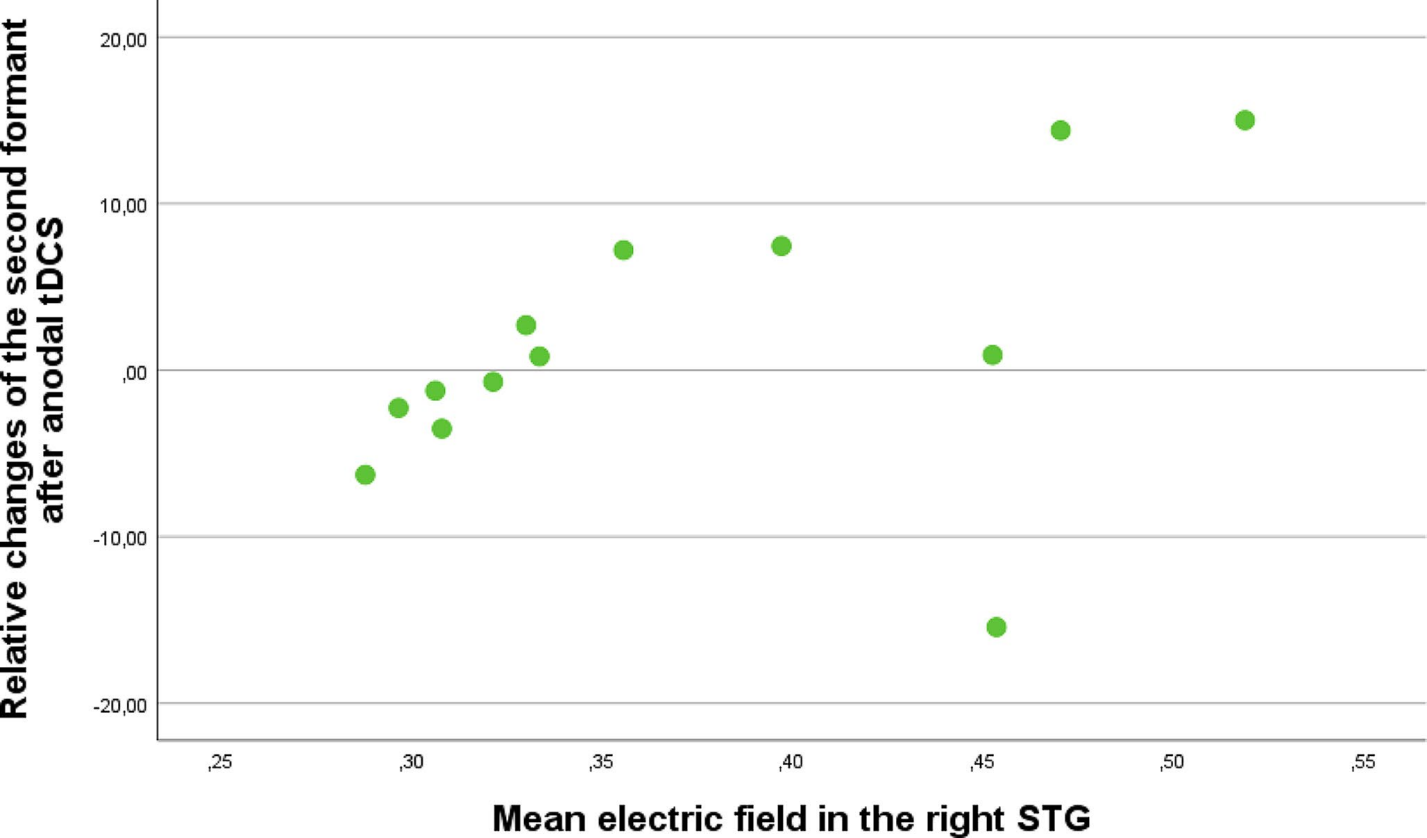
Correlation between the mean electric field in the right STG and relative changes in the standard deviation of the second formant after anodal tDCS

Electric field simulation, as assessed by SimNIBS, (Thielscher et al. 2015) indicated a significant correlation between the mean electric field in the right STG and relative changes in the standard deviation of the second formant after anodal tDCS (*R* = 0.637; *p* = 0.019) (see Table 5 and Fig. 2). This parameter describes the tongue and jaw movements, and it is used for evaluating articulation precision (Brabenec et al. 2017). However, this parameter did not significantly change due to the stimulation.

**Table 5:** Correlations between stimulated electric fields in the right STG and relative changes in acoustic parameters after stimulation

		relF1SD	relF2SD	relF0SD	SPIR	DurMED
Anodal tDCS	Spearman R	0.231	**0.637**	-0.192	-0.159	-0.450
	*P* value	0.448	**0.019**	0.529	0.603	0.123
Cathodal tDCS	Spearman R	0.165	0.033	0.099	-0.407	0.093
	*P* value	0.590	0.915	0.748	0.168	0.762

## Discussion

This exploratory cross-over randomized study found that anodal tDCS targeting the auditory feedback area in the right hemisphere can significantly improve motor speech fluency in PD patients. Moreover, the electric field in this region was positively correlated with anodal tDCS-induced changes in articulation precision.

These results are in partial accord with the results of our rTMS study in which low-frequency stimulation was used. Notably, rTMS-induced BOLD signal increases of the right STG were associated with changes of the same articulation parameter as in the current study (Brabenec et al. 2019). Therefore, it seems plausible that both stimulation protocols may lead to similar neural changes, but unlike in our rTMS study the effect of anodal tDCS alone was probably too weak to translate into significant behavioral improvements in articulation. Improved motor speech fluency was not identified in our single session rTMS study; however, there was a trend for improved speech rhythmicity; in other words, both stimulation protocols positively modulated temporal aspects of motor speech output. It has to be pointed out that different tasks were used in the two studies: reading of simple sentences in the rTMS study and reading of the whole paragraph in the current study.

This study has several limitations. We used the same MNI coordinates for all participants and did not implement individualized stimulation. However, it is important to note that tDCS stimulation is inherently less focal, and this pilot study served as a preparation for home-based stimulation, where achieving precise individualization is not practically feasible. Our sample size was determined based on the immediate medium aftereffects observed in a single session of rTMS (Brabenec et al. 2019), but the effects of tDCS could be much weaker. Nevertheless, we observed significant effect of tDCS on speech.

The main difference between the effects of rTMS and tDCS is that rTMS can trigger an action potential and tDCS can only modulate resting membrane potential and change the probability of the action potential occurrence (Nitsche et al. 2008). Thus, tDCS affects active neurons and is usually combined with behavioral training for more pronounced and long-term effects (Ni et al. 2022). In clinical practice, the gold standard for treatment of HD is currently the LSVT (Yuan et al. 2020). This high-effort speech therapy is primarily focused on improving speech loudness; previous studies showed that LSVT also increased the activation of the right STG and these changes correlated with improved speech intelligibility (Baumann et al. 2018).

Therefore, in a future study, we will investigate long-term behavioral effects and brain plasticity changes due to the home-based long-term tDCS as an add-on treatment to remote LSVT, delivered via telepractice.

## Data Availability

The data that support the findings of this study are available from the corresponding author upon reasonable request.
